# Efficacy and safety of oncolytic virus combined with chemotherapy or immune checkpoint inhibitors in solid tumor patients: A meta-analysis

**DOI:** 10.3389/fphar.2022.1023533

**Published:** 2022-11-14

**Authors:** Xiangxing Liu, Jiaojiao Zhang, Keqing Feng, Simin Wang, Liming Chen, Suping Niu, Qian Lu, Yi Fang

**Affiliations:** ^1^ Department of Clinical Pharmacy, Xuzhou Medical University, Xuzhou, China; ^2^ Department of Clinical Pharmacy, Ocean University of China, Qingdao, China; ^3^ Nursing Department, Peking University People’s Hospital, Beijing, China; ^4^ Clinical Trial Institution, Scientific Research Department, Peking University People’s Hospital, Beijing, China; ^5^ Jiangsu Key Laboratory of New Drug Research and Clinical Pharmacy, Xuzhou Medical University, Xuzhou, China; ^6^ Clinical Trial Institution, Peking University People’s Hospital, Beijing, China

**Keywords:** ICIs, oncolytic virus, oncolytic virus combination therapy, oncolytic virotherapy, single-arm meta-analysis

## Abstract

**Background:** In recent years, several clinical trials have focused on oncolytic virus (OVs) combined with chemotherapy or immune checkpoint inhibitors (ICIs) in solid tumor patients, which showed encouraging effects. However, few studies have concentrated on the summary on the safety and efficacy of the combined treatments. Therefore, we conducted this meta-analysis to explore the safety and curative effect of the combined therapy.

**Methods:** We searched the PubMed, Cochrane Library, Embase, and Clinicaltrials.gov databases to comprehensively select articles on OVs combined with chemotherapy or ICIs for the solid tumor treatment. Overall survival (OS), progression-free survival (PFS), 1-year survival rate, 2-year survival rate, objective response rate (ORR), and adverse events (AEs) were the outcomes.

**Results:** Fifteen studies with 903 patients were included in this meta-analysis. The pooled ORR was 32% [95% confidence interval (CI): 27–36%, I^2^ = 24.9%, *p* = 0.239]. Median OS and median PFS were 6.79 months (CI: 4.29–9.30, I^2^ = 62.9%, *p* = 0.044) and 3.40 months (CI: 2.59–4.22, I^2^ = 0.0%, *p* = 0.715), respectively. The 1-year survival rate was 38% (CI: 0.29–0.47, I^2^ = 62.9%, *p* = 0.044), and the 2-year survival rate was 24% (CI: 12–37%, I^2^ = 0.0%, *p* = 0.805). The most common AEs were fever (63%, CI: 57–69%, I^2^ = 2.3%, *p* = 0.402), fatigue (58%, CI: 51–65%, I^2^ = 49.2%, *p* = 0.096), chill (52%, CI: 43–60%, I^2^ = 0.0%, *p* = 0.958), and neutropenia (53%, CI: 47–60%, I^2^ = 0.0%, *p* = 0.944).

**Conclusion:** OVs combined with ICIs showed a better efficacy than OVs combined with chemotherapy, which lends support to further clinical trials of OVs combined with ICIs. In addition, OVs combined with pembrolizumab can exert increased safety and efficacy. The toxicity of grades ≥3 should be carefully monitored and observed. However, high-quality, large-scale clinical trials should be completed to further confirm the efficacy and safety of OVs combined with ICIs.

**Systematic Review Registration**: [https://www.crd.york.ac.uk/PROSPERO/login.php], identifier [RD42022348568].

## Introduction

Cancer is a leading cause of death and public health problems worldwide, requiring long-term collaboration between a large country and a large community. An estimated 19.3 million new cancer cases (18.1 million excluding non-melanoma skin cancer) and nearly 10.0 million cancer deaths (9.9 million excluding non-melanoma skin cancer) were reported in 2020 (Sung et al., 2021).

Chemotherapy is a traditional treatment for cancer. However, it often does not produce satisfactory results due to its cytotoxicity, low targeting, drug resistance, intolerable side effects, and high risk of recurrence (Garmaroudi et al., 2022). In recent years, immune checkpoint inhibitors (ICIs) have emerged as a cancer treatment. Unfortunately, ICIs monotherapy presents some challenges due to insufficient T-cell infiltration, defective antigen processing and presentation, and poor expression of programmed death receptors (such as PD-L1) (Zhou et al., 2022). One study reported an objective response rate (ORR) of 9% in ICI monotherapy (Zhu et al., 2021).

Oncolytic virus (OVs) therapy is expected to emerge as a method with great potential for solid tumor treatment. OV therapy began in 1904. A woman with chronic leukemia was reported to have unexpectedly improved after contracting influenza (Garmaroudi et al., 2022). OVs are divided into oncolytic RNA viruses and oncolytic DNA viruses. Oncolytic RNA viruses include reoviruses, paramyxoviruses, and picornaviruses. Oncolytic DNA viruses include the herpes viruses, adenoviruses, and poxviruses (Li Z et al., 2020). Globally, four OVs have been approved for cancer treatment. Following the approval of Talimogene Laherparepvec (T-VEC) by the U.S. Food and Drug Administration (FDA), a number of relevant clinical trials have been conducted. However, OV monotherapy has been less effective. It was reported that OV monotherapy had a 25% ORR, while OVs combined with ICIs had a 45% ORR for advanced melanoma treatment (Zou et al., 2020). OVs combined with chemotherapy (e.g., 5-fluorouracil, paclitaxel, doxorubicin, or cyclophosphamide) and ICIs (e.g., pembrolizumab or ipilimumab) are available, and an increasing number of clinical trials are being conducted (Puzanov et al., 2016; Villalona-Calero et al., 2016; Mahalingam et al., 2017; Bernstein et al., 2018; Eigl et al., 2018; Mahalingam et al., 2020; Puzanov et al., 2020). In some studies, OVs combined with chemotherapy and ICIs have demonstrated in vitro activity (Sei et al., 2009; Rajani et al., 2016). It has been reported that OVs combined with cisplatin and 5-fluorouracil in patients with recurrent head and neck cancer have achieved an 53% ORR (Khuri et al., 2000), and T-VEC combined with ipilimumab in previously untreated, unresectable stage IIIB-IV melanoma has achieved an 50% ORR (Puzanov et al., 2016). Several studies have confirmed that combining OVs with chemotherapy (e.g., gemcitabine, docetaxel, or carboplatin) or ICIs (e.g., pembrolizumab or ipilimumab) makes an encouraging efficacy and shows potential for development in further research (Khuri et al., 2000; Lu et al., 2004; Villalona-Calero et al., 2016; Cohn et al., 2017; Mahalingam et al., 2017; Kelly et al., 2020; Mahalingam et al., 2020; Soliman et al., 2021). Completed clinical trials also provided support to enhance therapeutic responses in the combined treatments (Harrington et al., 2020; Mahalingam et al., 2020; Puzanov et al., 2020; Bazan-Peregrino et al., 2021; Soliman et al., 2021).

A meta-analysis by Li Y et al. (2020) compared OVs combined with traditional treatment and traditional treatment alone in patients with cancer. However, we were unable to evaluate the effect of OVs combined with ICIs. Additionally, although the OV-chemo/immunotherapy combination has a positive therapeutic effect, its safety and efficacy deserve attention. Therefore, we carried out this study to assess the effects of combined OV therapy and hoped that the results we obtained could provide an available option for solid tumor treatment.

## Methods

### Literature search strategy

We searched studies in PubMed, Cochrane Library, and EMBASE. We searched up to 17 May, 2022. Studies published on ClinicalTrials.gov were screened as well. English was a language restriction. The following search terms were used: “oncolytic virus”, or “oncolytic virotherapy”, or “oncolytic virus combination therapy”, or “oncolytic virus combined with chemotherapy”, or “oncolytic virus combined with ICIs therapy”, or “oncolytic virus combined with pembrolizumab or nivolumab or ipilimumab”. The included studies were conditionally filtered, and relevant articles on the subject were reviewed, while those that were not on the subject were excluded. The study was conducted according to Preferred Reporting Item for Systematic Reviews and Meta-Analyses (PRISMA) (Page et al., 2021) and was registered in the PROSPERO database (CRD42022348568).

### Inclusion and exclusion criteria

The eligible studies: 1) randomized controlled trials (RCTs) or single-arm clinical trials with OVs combined chemotherapy or ICIs treatments in solid tumor patients were selected; 2) the studies reported at least one of the following outcomes: objective response rate (ORR), progression-free survival (PFS), overall survival (OS), 1-year survival rate, 2-year survival rate, or adverse events (AEs) of incidence ≥50% or grade ≥3; 3) studies that published the most current version and data were also included; 4) for RCTs, the experimental group comprised patients treated with OVs combined with chemotherapy or immunotherapy, while the control group comprised patients treated with chemotherapy or immunotherapy alone. For single-arm trials, the experimental group was treated with OVs combined with chemotherapy or ICIs; 5) the confidence interval (CI) of the aggregated data was set at 95%.

The exclusion criteria were as follows: 1) case reports, letters, reviews, conference abstracts, animal studies, and *in vitro* studies were unselected; 2) studies that published without English language were excluded; and 3) studies with overlapping or duplicated data and studies with the same identical clinical trial numbers were also removed.

### Quality assessment

The Cochrane risk of bias tool was used to assess the quality of the RCTs. Seven items (random sequence generation (selection bias), allocation concealment (selection bias), blinding of participants and personnel (performance bias), blinding of outcome assessment (detection bias), incomplete outcome data (attrition bias), selective reporting (reporting bias), and other bias were included in the Cochrane risk of bias tool. Each entry was divided into high risk, low risk, and unclear risk. An element was identified as having an unclear risk of bias if it did not contain enough information. For sing arm trials, Risk of Bias in Non-randomized Studies-of Interventions (ROBINS-I) was used (Morche et al., 2020).

In the sensitivity analysis, a single study was excluded, and the meta-analysis was reconducted to assess the comprehensive effect. Publication bias was tested using funnel plots. Publication bias was not necessary to perform if there were  <10 included studies (Li Y et al., 2020).

### Data extraction

The full-text screens of the included studies and data extraction were performed independently by two investigators. Any differences of opinion were resolved by a third investigator. First author, year, publication, race, combination therapy, age, clinical phase, injection mode, total number of patients, timing of follow-up, types of cancer, and clinical endpoints were extracted. Additional documents or appendices to the included studies were also carefully reviewed.

### Statistical analysis

In this study, RevMan 5.3 was applied to assess the quality of RCTs, and Stata version 14 was used for statistical analysis. Pooled ORR, OS, PFS, 1-year survival rate, 2-year survival rate, and AEs were summarized. Heterogenicity among studies was assessed using the chi-squared test and I^2^. A fixed-effects model was applied if the heterogenicity I^2^ < 50% or *p* > 0.05. Otherwise, the random-effects model was used (Chen and Benedetti, 2017). Additionally, high heterogeneity was adjusted by subgroup analysis. Begg’s test and Egger’s test were used to precisely assess the publication bias of eligible studies (Higgins, 2003).

## Results

### Literature search and study selection

**FIGURE 1:**
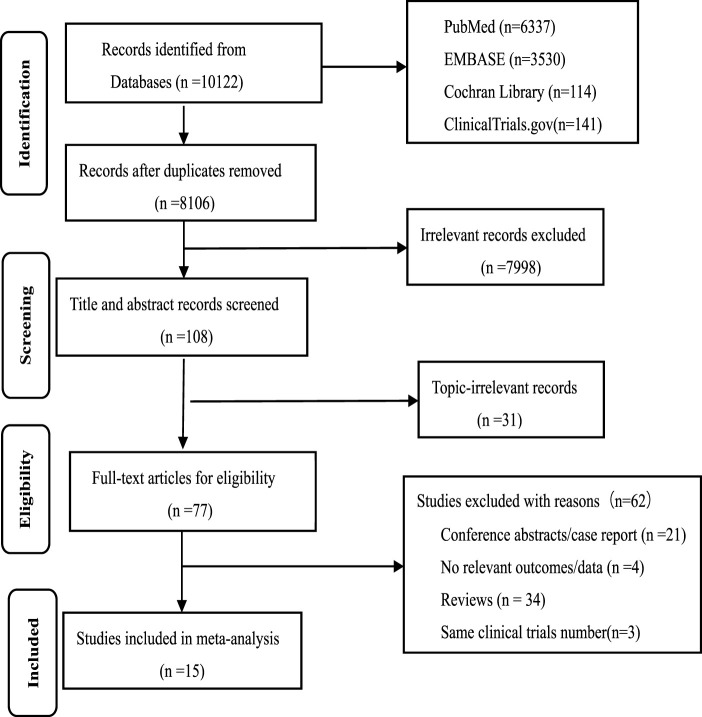
Flow chart of the study selection process.

In total, 10,122 records were identified in PubMed, Cochrane Library, EMBASE and ClinicalTrials.gov. Then 108 records were retained following deletion of duplicates and irrelevant records. Of these, 77 references were retained following the selection of the titles and summaries, and 62 studies were deleted for following reasons: reviews, conference summaries, case report, irrelevant outcomes, and same clinical trial number. Finally, 15 prospective clinical trials with 903 patients were incorporated into this meta-analysis. Six were RCTs, and nine were single-arm trials. Details on the study screening and selection process can be found in Figure 1.

### Characteristics of included studies

The major features of these studies are presented in Table 1. All included studies were released by 17 May 2022.Four OVs were included: herpes simplex virus type 1(HSV-1) (*n* = 4), reovirus (*n* = 8), vaccinia virus (*n* = 1), and adenovirus (*n* = 2). The types of tumors were various. The races were White, African American, Caucasian, Hispanic, Asian, and Black.

**TABLE 1:** Main characteristics of included studies.

Study	N	Combination therapy	Tumor type	Injection mode	Races	Trials type
Soliman et al. (2021)	9	T-VEC + paclitaxel and doxorubicin/cyclophosphamide	Breast cancer	IT	White, African American, other	Single-arm
Mahalingam et al. (2020)	11	Pelareorep + pembrolizumab, 5-fluorouracil, gemcitabine, or irinotecan	Pancreatic adenocarcinoma	IV	Hispanic/Non-Hispanic	Single-arm
Puzanov et al. (2020)	198	T-VEC + ipilimumab	Melanoma	IT	White	RCT
Harrington et al. (2020)	36	T-VEC + pembrolizumab	Squamous cell carcinoma of the head and neck (R/M HNSCC)	IT	Hispanic or Latino/Non-Hispanic or Latino	Single-arm
Kelly et al. (2020)	20	T-VEC + pembrolizumab	Advanced or metastatic sarcoma	IT	White	Single-arm
Moehler et al. (2019)	129	Pexa-Vec (JX-594) + best supportive care (BSC)	Advanced hepatocellular carcinoma	IT	Asian, White	RCT
Bernstein et al. (2018)	74	Pelareorep + paclitaxel	Metastatic breast cancer	IV	Canada	RCT
Mahalingam et al. (2018)	34	Pelareorep + gemcitabine	Advanced pancreatic adenocarcinoma	IV	Caucasian, Black, Hispanic, Asian	Single-arm
Eigl et al. (2018)	85	Pelareorep + docetaxel	Metastatic castration resistant prostate cancer	IV	Canada	RCT
Mahalingam et al. (2017)	14	Pelareorep + carboplatin and paclitaxel	Advanced malignant melanoma	IV	Caucasian/Non-Hispanic/Hispanic	Single-arm
Cohn et al. (2017)	100	Pelareorep + paclitaxel	Recurrent ovarian, tubal, or peritoneal cancer	IV	White	RCT
Villalona-Calero et al. (2016)	37	Pelareorep + paclitaxel and carboplatin	Metastatic or recurrent non-small cell lung cancer	IV	White, African American	Single-arm
Anne et al. (2016)	73	Pelareorep + carboplatin and paclitaxel	Metastatic pancreatic adenocarcinoma	IV	African American, Caucasian	RCT
Lu et al. (2004)	46	H101 + chemotherapy, H101	Advanced cancers	IT	Asian	Single-arm
Khuri et al. (2000)	37	ONYX-015 + cisplatin and 5-fluorouracil	Recurrent head and neck cancer	IT	NA	Single-arm

NA= unknown. RCT, randomized controlled trial. IT, intratumoral. IV, intravenous. T-VEC, Talimogene Laherparepvec, herpes simplex virus type (HSV-1); Pelareoprep, reovirus; H101 and ONYX-015, adenovirus; Pexa-Vec virus (JX-594), vaccina virus.

### Quality assessment

In our assessment, some RCTs incorporated random allocation performed and the use of the random sequence generation method. Non-blinding had no significant effect on literature quality evaluation and therefore was considered as a low-risk factor. For single-arm trials, most of the assessment criteria showed a low risk of bias (Supplementary Material).

## Publication bias

Assessment of the pooled ORR did not identify significant publication bias among the included studies (*p* = 0.725 for Egger’s test and *p* = 0.805 for Begg’s test). The funnel plot is shown in Supplementary Material.

### Efficacy

#### Objective response rate

**FIGURE 2:**
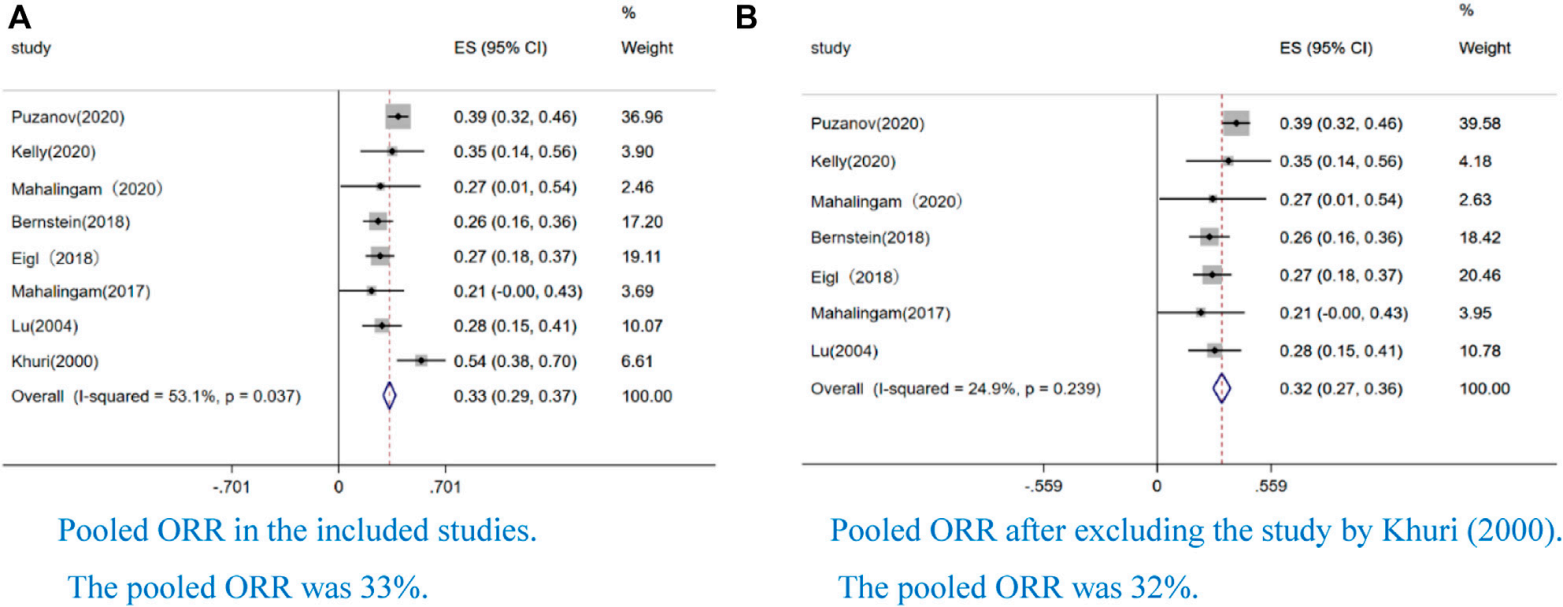
Forest plot of the pooled rate of objective response (ORR) to OVs combined with chemotherapy or ICIs. Eight studies reported ORR. We performed a sensitivity analysis because we found that the pooled ORR has significant heterogeneity (I2=53.1%, p=0.037) (Figure 2). After excluding the study by Khuri et al, we finally obtained a pooled ORR of 32% (CI: 27–36%, I2=24.9%, p = 0.239) (Figure 2). In the all forest plots that we presented, the square represents effect sizes from a single study and the side represent weight; the diamond represents the pooled result of single study; the red dotted line represents the mean of the pooled data; the horizontal line represents a single study; the horizontal line length represents the 95% confidence interval (CI) of the effect size of a single study; the solid line perpendicular to the X-axis represents the null line and the coordinate of the effect value is 0 (continuous variable); I^2^ > 50% indicated that the pooled data was highly heterogeneous; weight represent the proportion of single study.

Eight studies reported an ORR. We performed a sensitivity analysis because we found that the pooled ORR had significant heterogeneity (I^2^ = 53.1%, *p* = 0.037) (Figure 2A). After excluding the study by Khuri et al., we finally demonstrated a pooled ORR of 32% (CI: 27–36%, I^2^ = 24.9%, *p* = 0.239) (Figure 2B), and the result indicated that the pooled ORR was reliable the study by Khuri et al.

#### Survival

**FIGURE 3:**
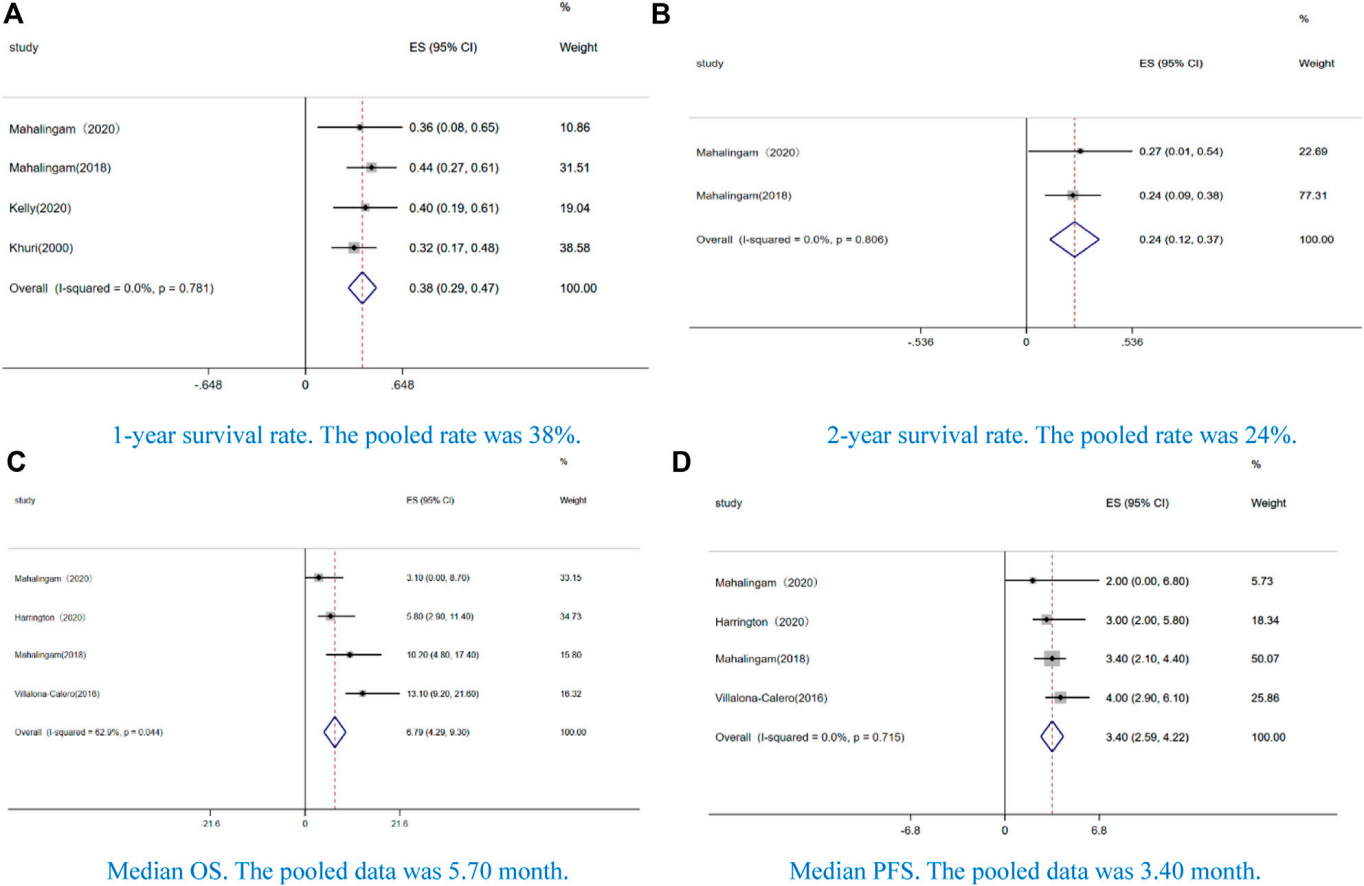
Forest plot of the pooled data of survival, including **(A)** 1-year survival rate, **(B)** 2-year survival rate, **(C)** median overall survival (OS), and **(D)** median progression-free survival (PFS).

Four studies reported 1-year survival rate (38%, CI: 0.29–0.47, I^2^ = 0.0%, *p* = 0.000) (Figure 3A), and two studies reported 2-year survival rate (24%, CI: 12–37%, I^2^ = 0.0%, *p* = 0.805) (Figure 3B). The median OS was 6.79 months (CI: 4.29–9.30, I^2^ = 62.9%, *p* = 0.044) (Figure 3C), and the median PFS was 3.40 months (CI: 2.59–4.22, I^2^ = 0.0%, *p* = 0.715) (Figure 3D).

#### Toxicities

**FIGURE 4:**
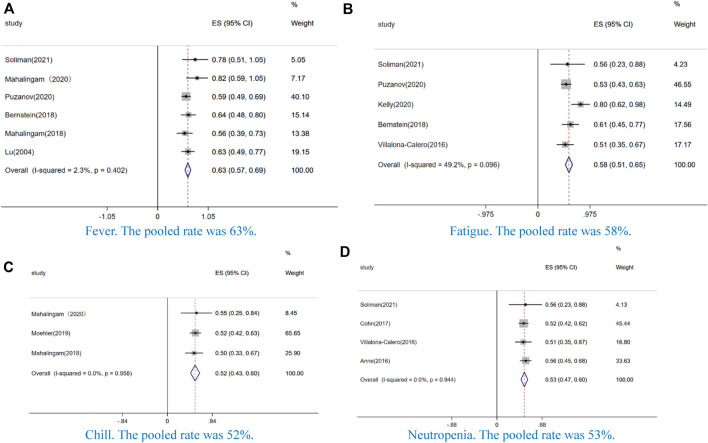
Forest plot of the pooled data that AE incidence ≥50%, including **(A)** fever, **(B)** fatigue, **(C)** chill, and **(D)** neutropenia.

**FIGURE 5:**
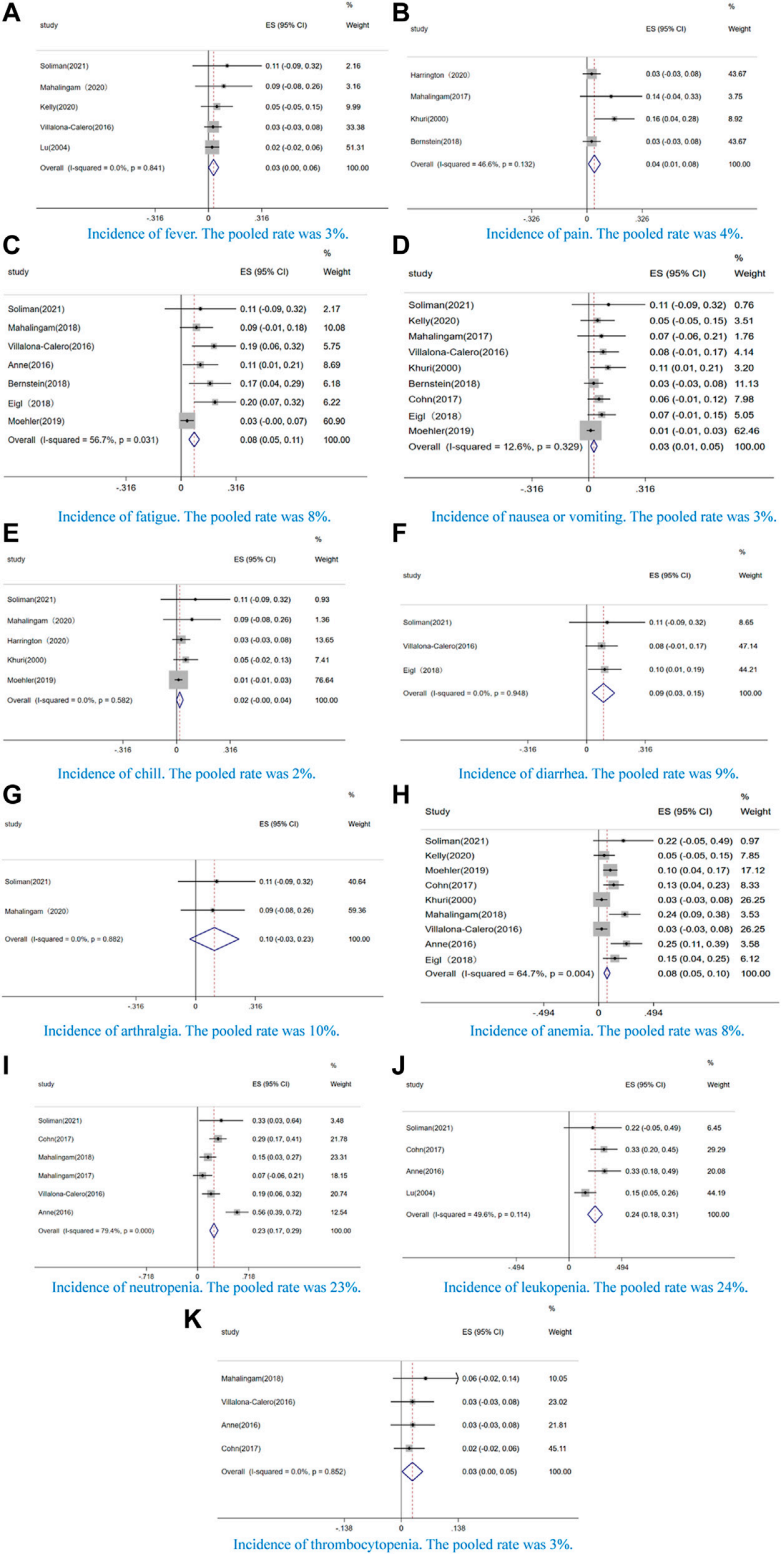
Forest plot of the pooled data that the incidence of incidence of grade 3 or greater AEs, including **(A)** fever, **(B)** pain, **(C)** fatigue, **(D)** nausea or vomiting, **(E)** chill, **(F)** diarrhea, **(G)** arthralgia, **(H)** anemia, **(I)** neutropenia, **(J)** leukopenia, and **(K)** thrombocytopenia.

In our study, the AEs of incidence ≥50% were fever (63%, CI: 57–69%, I^2^ = 2.3%, *p* = 0.402) (Figure 4A), fatigue (58%, CI: 51–65%, I^2^ = 49.2%, *p* = 0.096) (Figure 4B), chill (52%, CI: 43–60%, I^2^ = 0.0%, *p* = 0.958) (Figure 4C), and neutropenia (53%, CI: 47–60%, I^2^ = 0.0%, *p* = 0.944) (Figure 4D). Additionally, we analyzed the AEs of grade ≥ 3. Among these AEs, non-hematological toxicity such as fever (3%, CI: 0–6%, I^2^ = 0.0%, *p* = 0.841) (Figure 5A), pain(4%, CI: 1–8%, I^2^ = 46.6%, *p* = 0.132) (Figure 5B), fatigue (8%, CI:5–11%, I^2^ = 56.7%, *p* = 0.031) (Figure 5C), nausea or vomiting (4%, CI:2–6%, I^2^ = 12.6%, *p* = 0.329) (Figure 5D), chill (2%, CI:0–4%, I^2^ = 0.0%, *p* = 0.582) (Figure 5E), diarrhea (9%, CI:3–15%, I^2^ = 0.0%, *p* = 0.948) (Figure 5F), and arthralgia (10%, CI:3–23%, I^2^ = 0.0%, *p* = 0.882) (Figure 5G) and hematological toxicity such as anemia (8%, CI: 5–10%, I^2^ = 64.7%, *p* = 0.004) (Figure 5H), neutropenia (23%, CI:17–29%, I^2^ = 79.4%, *p* = 0.000) (Figure 5I), leukopenia (26%, CI:16–36%, I^2^ = 49.6%, *p* = 0.114) (Figure 5J), and thrombocytopenia (3%, CI:0–5%, I^2^ = 0.0%, *p* = 0.852) (Figure 5K) were observed.

#### Subgroup analyses

**FIGURE 6:**
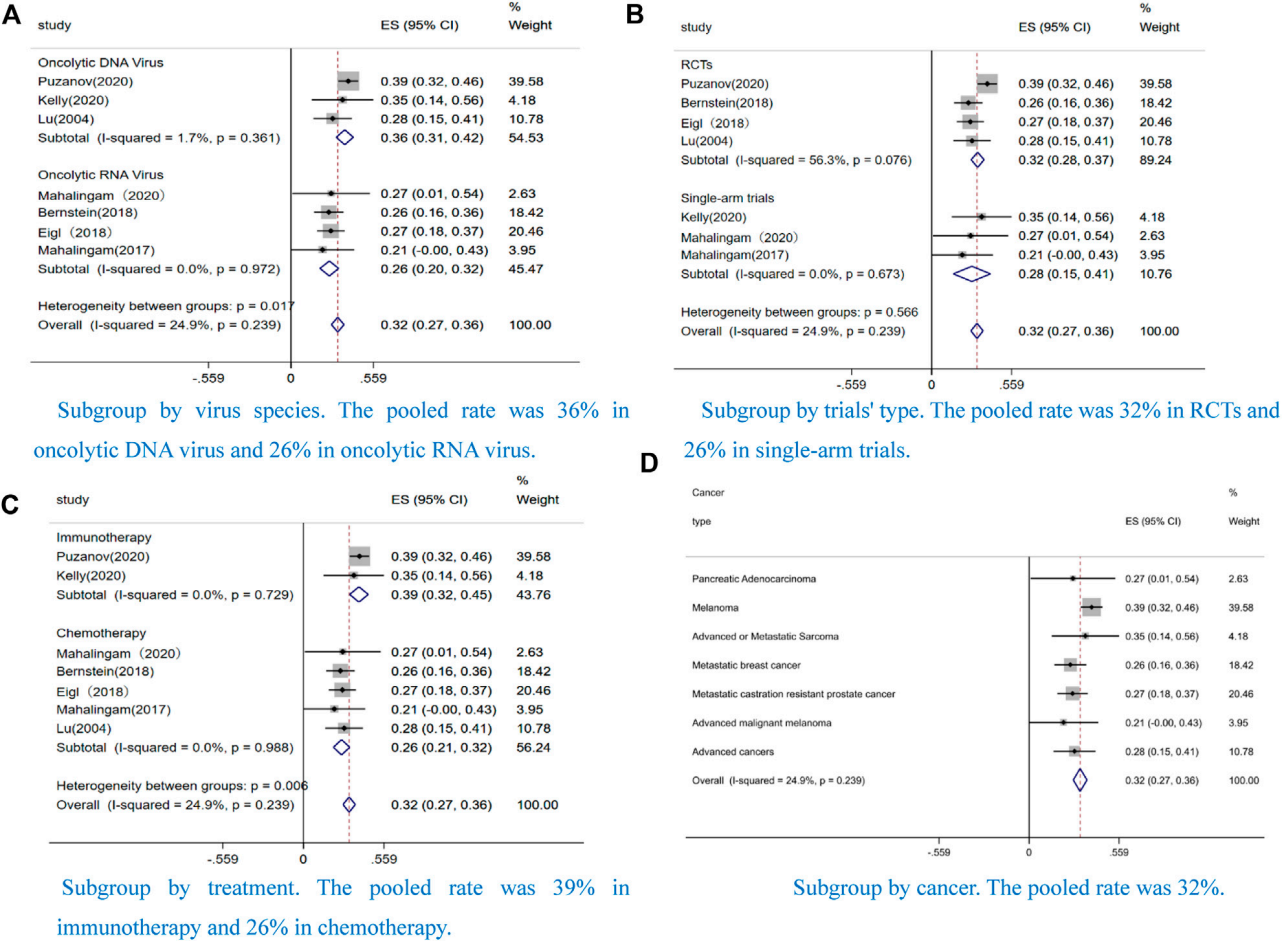
Forest plot of the pooled objective response rates (ORRs) in different subgroups, including **(A)** Subgroup by virus species **(B)** Subgroup by trials' type **(C)** Subgroup by treatment **(D)** Subgroup by cancer.

We conducted a pooled ORR of 26% (CI: 20–32%, I^2^ = 0.0%, *p* = 0.972) in the oncolytic RNA virus and 36% (CI: 31–42%, I^2^ = 1.7%, *p* = 0.361) (Figure 6A) in the oncolytic DNA virus. We also demonstrated a pooled ORR of 32% (CI: 28–37%, I^2^ = 56.3%, *p* = 0.076) in RCTs and 28% (CI: 15–41%, I^2^ = 0.0%, *p* = 0.673) (Figure 6B) in single-arm trials. Additionally, we performed a subgroup analysis according to treatment. The pooled ORR was 39% (CI: 32–45%, I^2^ = 0.0%, *p* = 0.729) in OVs combined ICIs and 26% (CI: 21–32%, I^2^ = 0.0%, *p* = 0.988) (Figure 6C) in OVs combined chemotherapy. Subgroup analysis by type of cancer was also performed (32% ORR, CI: 27–36%, I^2^ = 24.9%, *p* = 0.239) (Figure 6D). The result was consistent with the pooled ORR mentioned above (Figure 2B). We observed a low heterogenicity in the pooled data on age (Supplementary Material).

**FIGURE 7:**
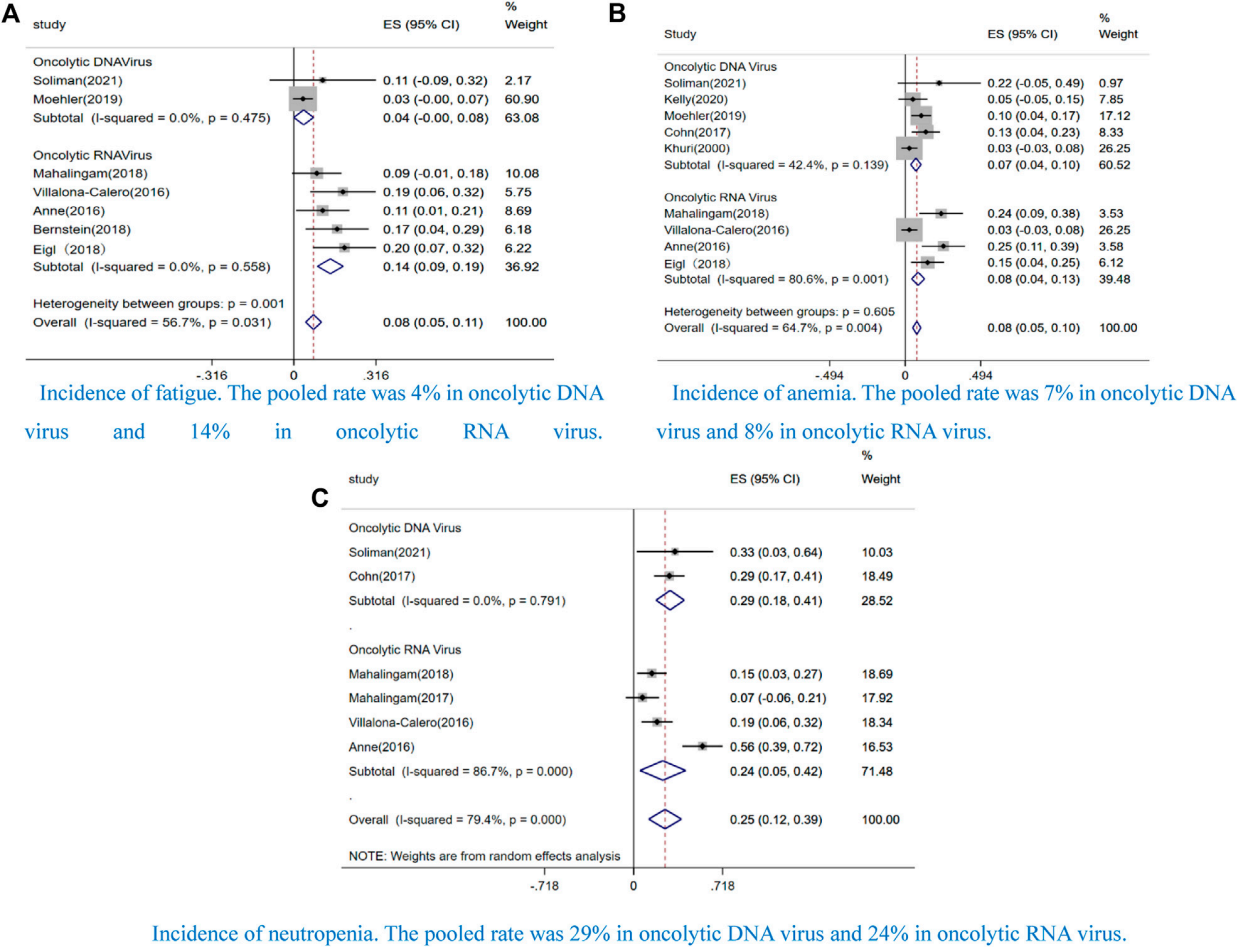
Forest plot of the pooled rate by virus species that incidence of grade 3 or greater AEs, including **(A)** fatigue, **(B)** anemia, and **(C)** neutropenia.

The incidence of grade ≥ 3 fatigue was 4% (CI: 0.0–8%, I^2^ = 0.0%, *p* = 0.475) in the oncolytic DNA virus and 14% (CI: 9–19%, I^2^ = 0.0%, *p* = 0.558) in the oncolytic RNA virus (Figure 7A), the incidence of grade ≥ 3 anemia was 7% (CI: 3–13%, I^2^ = 42.4%, *p* = 0.139) in the oncolytic DNA virus and 8% (CI: 4–13%, I^2^ = 80.6%, *p* = 0.001) in the oncolytic RNA virus (Figure 7B), and the incidence of grade ≥ 3 neutropenia was 29% (CI: 18–41%, I^2^ = 0.0%, *p* = 0.791) in the oncolytic DNA virus and 24% (CI: 5–42%, I^2^ = 88.7%, *p* = 0.000) in the oncolytic RNA virus (Figure 7C).

## Discussion

In this meta-analysis, we explore the efficacy and safety of OV-chemo/immunotherapy in multiple solid tumors. Compared with other meta-analysis (Li Y et al., 2020; Li Z et al., 2020; Zou et al., 2020), we included the single-arm trials, collecting as much data as possible on combination therapy with OVs and minimizing potential bias.

Compared with the meta-analysis by Zou et al. (2020), we demonstrated a lower ORR in the treatment of OVs combined with ICIs [39% (CI; 32–45%) *vs.* 45% (CI: 38–52%), *p *= 0.218]. One explanation on the difference might be that we excluded the data from abstract and conference, resulting to less pooled data. In addition, in our study, the ORR was no significant difference between RCTs and single-arm trials. Notably, we found that the application of the oncolytic DNA virus showed a better ORR than the oncolytic RNA virus. We thought one reason for this may have been related to the injection method of the OVs. Innate immunity is a major obstacle in OVs application. In our present meta-analysis, Pelareorep (oncolytic RNA virus) was delivered via intravenous injection, while T-VEC, Pexa-Vec (JX-594), H101, and ONYX-015 (oncolytic DNA virus) were administered via intratumor injection. We believed that intravenous injection increases OV exposure, making it more likely to occur anti-viral response. Earlier studies confirmed that the responses to anti-viral played an important role in inducing anti-tumor response, which enhanced clinical efficacy (White et al., 2008). Unlike intravenous injection, intratumor injection effectively targeted OVs at the tumor site and reduced exposure, thus preventing anti-viral responses. Although OVs may increase the risk of infecting tumor cells and enhance anti-tumor effect, anti-viral responses may still be an obstacle. Previous study by Power et al. (Power et al., 2007) came up with an idea that using tumor cells as OV carrier. Carrier cells were targeted at the tumor site and then infected tumor cells by OV replication and lysing, which exerted the maximum effect and effectively avoided anti-viral response. Another study reported that OVs improved the efficacy of CAR-T cell therapy (Zarezadeh Mehrabadi et al., 2022), which suggested a potential method for cancer treatment. Recently, a clinical trial on binary oncolytic adenovirus in combination with HER2-specific autologous CAR VST, advanced HER2 positive solid tumors has been in process (NCT03740256), which might provide more supporting evidence in the OV combination.

Another important finding was that OVs combined with ICIs showed a better ORR than OVs combined with chemotherapy. Recently, ICIs have come to be regarded as a standard of care in malignant tumor (Minichsdorfer et al., 2022). However, only a small number of patients respond to ICIs therapy, while many patients were primarily resistant to ICIs (Ribas et al., 2017). ICI monotherapy is now moving toward combination therapy. Encouragingly, ICIs combined with OVs therapy have been shown to provide superior therapeutic outcomes (Zhou et al., 2022). In the meta-analysis conducted by Zou et al. (2020), it was concluded that the ORR could be improved to 45% if OVs are combined with ICIs for advanced melanoma. Lysing the host cells through self-replication forms a direct effect of OVs (Achard et al., 2018). Destroying the tumor microenvironment (TME) and inducing anti-tumor response are the major mechanisms of OVs (Bai et al., 2019; Wang et al., 2022). With these mechanisms, OVs lends a support for ICIs therapy by upregulating PD-L1 of tumor cells (Zhou et al., 2022). Notably, timing the ICIs combined with OVs is critical to achieving optimal outcomes. One study by Liu et al. (2017) verified that simultaneously administrating OVs and ICIs showed a better effect. However, good efficacy might be achieved if OVs are administered first (Zheng et al., 2019). OVs are cleared prematurely if the ICIs are administered first and it would be too challenging to be efficient if ICIs administered too late. In several studies, it has been reported that OVs combined with chemotherapy did not achieve desired results (Noonan et al., 2016; Eigl et al., 2018), while in other studies, the combined therapy reached satisfactory outcomes (Cohn et al., 2017; Mahalingam et al., 2017; Bernstein et al., 2018; Soliman et al., 2021). These differences might be related to the disease state of patients. KRAS mutation is common in some solid tumors (e.g., pancreatic cancer, lung cancer, and colon cancer), which leads to a disappointing prognosis. In the study by Noonan et al., KRAS (Kirsten rat sarcoma viral oncogene homolog) mutation was observed in most patients.

In the sensitivity analysis, we excluded each study from the results one by one and finally culled the study by Khuri et al. (2000). One reason for this was that the tumors in patients were all injected, which led to potential selection bias. There was no high heterogeneity in median PFS, 1-year survival rate, and 2-year survival rate in terms of curative effect. High heterogenicity was observed in pooled median OS. One reason might that we included too narrow a range of studies. In addition, dosage, interval, and period of OV administration might also be a source of heterogeneity. Tumor tropism, immunogenicity, and OV species could have affected efficacy.

Regarding safety, we found AEs of incidence ≥50% included fever, fatigue, chill, and neutropenia. AEs of incidences ≥10% and grade ≥3 included fatigue, arthralgia, anemia neutropenia, and leukopenia. It is clear that the incidence of hematological toxicity was higher than non-hematological toxicity. In the meta-analysis conducted by Li et al. (2020), it reported that OVs combined with chemotherapy had a significantly higher incidence of grade ≥ 3 adverse effects than chemotherapy alone. However, the authors note that this conclusion may have been false positive, and more studies are needed to determine this. Further, in the meta-analysis, an incidence of grade ≥3 anemia, thrombocytopenia, fatigue, influenza-like illness, and gastrointestinal adverse effects showed no significantly differences between the experimental groups and control groups. However, the incidence of grade ≥3 neutropenia showed a significant difference between the experimental groups and control groups. Therefore, we thought that neutropenia might be relevant to OVs. In a study conducted by Barnes et al. (2017), the incidence of anemia, thrombocytopenia, and neutropenia were also observed in cytotoxic chemotherapy. Although we observed that toxicity was partially increased in OVs combined with chemotherapy for solid tumor treatment, the results were consistent with the meta-analysis conducted by Li et al. Notably, some AEs that occurred in studies outside our scope should be noted. A fatal arterial hemorrhage was reported in the combination of OVs and pembrolizumab for recurrent or metastatic squamous cell carcinoma of the head and neck treatment (Harrington et al., 2020). In this study, the incidence of AEs of any grade was 55.6% and any grade≥ 3 was 13.9%, indicating that OVs combined with pembrolizumab might be a potential combination in solid tumor treatments. It is noteworthy that some AEs, such as influenza-like illness, pyrexia, peripheral neuropathy, pneumonia, septicemia, hypertension, neutropenic fever, and serious respiratory adverse reactions might occur in the combined therapy (Eigl et al., 2018; Harrington et al., 2020; Kelly et al., 2020). Finally, hematological toxicity in the combined therapy should be carefully monitored and observed.

Searching in ClinicalTrials.gov, we found some clinical trials on OVs (e.g., Seneca Valley virus, recombinant vaccinia GM-CSF, reovirus, herpes simplex virus, oncolytic measles virus, oncolytic adenovirus, and Newcastle virus) were combined with chemotherapy or ICIs in solid tumor treatment (e.g., pancreatic cancer, lung cancer, glioblastoma, brain cancer, and advanced bladder carcinoma) are in recruitment. However, we found that there have been few clinical trials on OVs treated for hematological disease. One phase I trial study examined the side effects and best dose of wild-type reovirus (pelareorep) when given together with dexamethasone, carfilzomib, and nivolumab in treating patients with multiple myeloma is active (NCT03605719). It has been reported that improved prognosis was achieved using OVs combined with chemotherapy or ICIs in hematological disease, including multiple myeloma (MM), acute myeloid leukemia (AML), chronic myeloid leukemia (CML), and chronic lymphocytic leukemia (CLL) (Innao, 2021). As these clinical trials proceed and their data are published, we believe that the efficacy and safety of OV combination therapy will be fully validated.

This meta-analysis had several strengths. First, we include single arm trials to comprehensively explore the efficacy and safety of combination therapy with OVs. Second, we included and analyzed the most recent versions of the results and previously unpublished data to prevent potential mistakes. Third, the outcomes of OS, PFS, 1-year survival rate, and 2-year survival rate could be assessed, which could not be done in other meta-analyses. To analyze the efficacy, we conducted the ORR according to treatments, types of OVs, and clinical trials. In terms of safety, we pooled data of incidence ≥50% AEs and grade ≥3AEs. In addition, we performed a subgroup analysis according to hematological and non-hematological toxicity. Previous meta-analyses (Li Y et al., 2020; Li Z et al., 2020; Zou et al., 2020; Xie et al., 2021) have only investigated a series of pairwise comparisons, so they failed to make comparisons without eligible clinical trials. In this study, we included not only RCTs but also selected single-arm trials and therefore gathered together comprehensive data. Although we could not identify whether the combination treatment was better than OV monotherapy, chemotherapy monotherapy, or ICI monotherapy as the study was based on solitary comparisons, we compared the difference with other meta-analysis based on RCTs.

The limitations of this study should be mentioned. First, we cannot identify whether combination treatment was better than OV monotherapy, chemotherapy monotherapy or ICIs monotherapy, as the study was based on solitary comparisons. Moreover, we cannot determine the safety profile of combination therapy is better than OVs monotherapy. Second, we did not consider types of tumor due to the insufficient number of prospective studies analyzing the same cancer. Additionally, we did not perform a group analysis for sex, race, or dose due to the insufficiency of the data. Third, few studies have provided relevant data such as that on OS, PFS, 1-year survival rate, and 2-year survival rate, which may have created an insufficiently convincing result. Finally, among the 15 studies, 2 were identified as phase I, and 13 were identified as phase II. No phase III clinical trials were included. Many clinical trials on OVs are still ongoing. Thus, these results could change if further studies are completed.

## Conclusion

We observed a high incidence of grade ≥3 hematological toxicity in OVs combination therapy, for example, neutropenia, anemia, and thrombocytopenia, which is consistent with the study by Li et al. We thought OVs combined with ICIs could be a potential approach in the solid tumor treatment. Although hematological toxicity was observed, the combined therapy demonstrated encouraging efficacy. As only a few randomized controlled clinical studies were involved in OVs combined with ICIs, more high-quality and large-scale studies are required to further evaluate the efficacy and safety. We believe that as the number of studies increases, OVs combined with ICIs will play an increasingly important role in solid tumor treatment.

## Data Availability

The original contributions presented in the study are included in the article/Supplementary Material. Further inquiries can be directed to the corresponding author.

## Abbreviations

AEs, adverse events; AML, acute myeloid leukemia; CLL, chronic lymphocytic leukemia; CML, chronic myeloid leukemia; DCs, dendritic cells; ICIs, immune checkpoint inhibitors; MM, multiple myeloma; NKs, natural-killer cells; KRAS, Kirsten rat sarcoma viral oncogene homolog; ORR, objective response rate; OS, overall survival; OVs, oncolytic virus; PFS, progression-free survival; RCTs, randomized clinical trials; TME, tumor microenvironment.
