# Supplementary material for: Efficacy and safety of oncolytic virus combined with chemotherapy or immune checkpoint inhibitors in solid tumor patients: A meta-analysis
Source: Front Pharmacol. 2022 Nov 14;13:1023533. doi: 10.3389/fphar.2022.1023533 (PMC9702820; doi:10.3389/fphar.2022.1023533)
Supplement: Supplementary file 1 [file DataSheet1.docx]

Supplementary 1 Forest plot of 1-year survival rate (subgroup by virus species). In the all forest plot that we presented, the square represent effect sizes from a single study and the side represent weight; the diamond represents the pooled result of single study; the red dotted line represents the mean of the pooled data; the horizontal line represents a single study; the horizontal line length represents the 95% confidence interval of the effect size of a single study; the solid line perpendicular to the X-axis represents the null line and the coordinate of the effect value is 0 (continuous variable); I-squared＞50% indicated that the pooled data was highly heterogeneous; weight represent the proportion of single study.In the pooled data, 1-year survival rate was 42% in oncolytic RNA virus and 35% in oncolytic DNA virus.


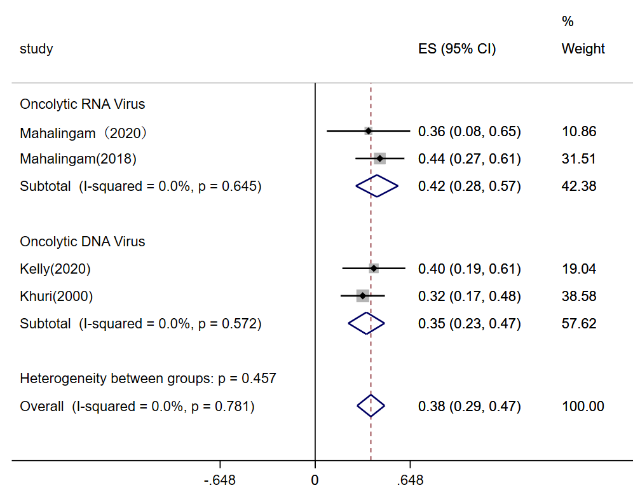


Supplementary 2 Pooled data in age. In the forest plot, the Average age is 60.25 years old. I-square =0%, suggesting that there was no heterogeneity.


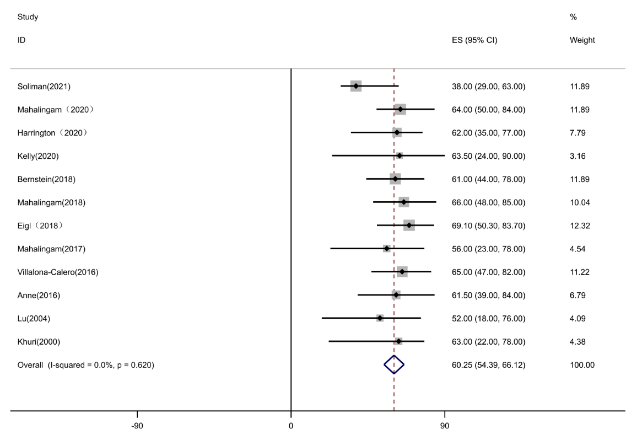


Supplementary 3 Funnel plot in age. Funnel plots can be used to identify publication or other biases, and at least 10 studies need to be included. If there are less than 10studies, the test power will be insufficient, which is difficult to evaluate the symmetry of the funnel plot. We did not perform other funnel plots because of the insufficient studies. The horizontal axis represents the effect size. The younger the age, the farther the point of the study is to the left; the older the age, the farther the point is to the right. The vertical axis is the standard error, the larger the sample size, the smaller the standard error and the more upward the distribution. The two diagonal lines are the 95% confidence intervals of the funnel plot. If studies are outside the scope, it suggests that it may be heterogeneity. In this figure, funnel plot is basically symmetrical, which suggested the low publication bias.


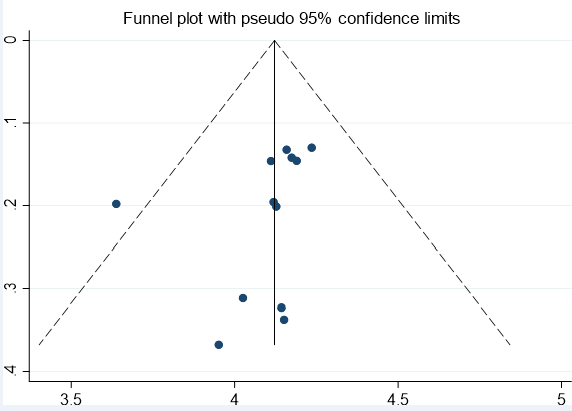


Supplementary 4 Risk of bias Assessment in single-arm clinical trials. For single-arm trials, most of the items are low-risk bias.


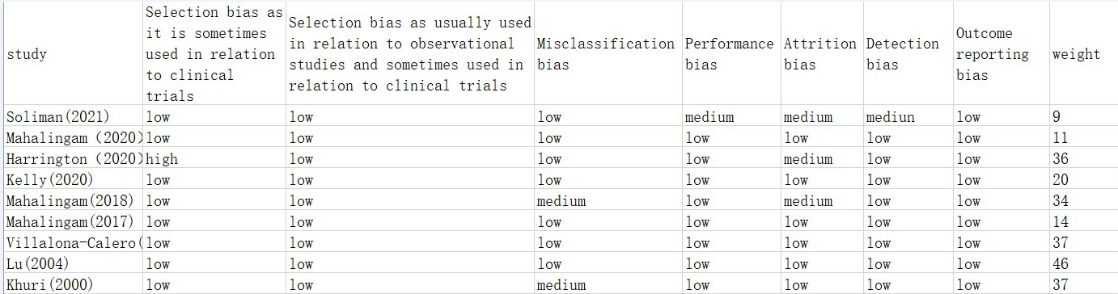


Supplementary 5 Risk of Bias Assessment in RCTs, including (a) Risk of bias graph in RCTs and (b)Risk of bias summary in RCTs. In our assessment, some RCTs mentioned random allocation performed and the use of the random sequence generation method. Non-blinding had no significant effect in literature quality evaluation and therefore was considered as a low-risk factor.


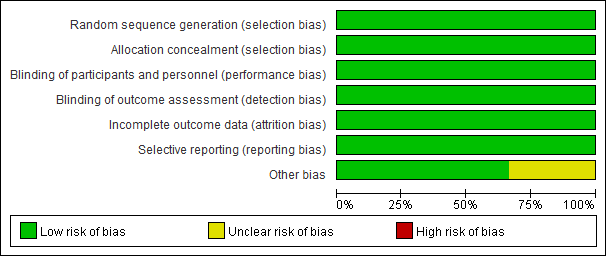
(a) Risk of bias graph in RCTs


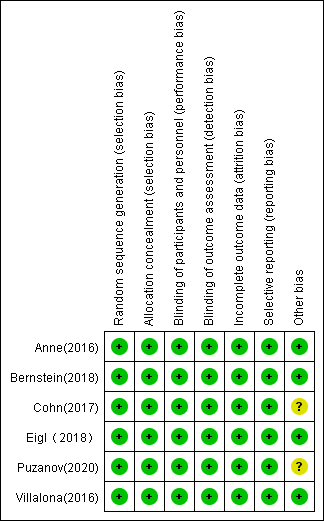


(b)Risk of bias summary in RCTs
